# The Transcription Factor Hobit Identifies Human Cytotoxic CD4^+^ T Cells

**DOI:** 10.3389/fimmu.2017.00325

**Published:** 2017-03-24

**Authors:** Anna E. Oja, Felipe A. Vieira Braga, Ester B. M. Remmerswaal, Natasja A. M. Kragten, Kirsten M. L. Hertoghs, Jianmin Zuo, Paul A. Moss, René A. W. van Lier, Klaas P. J. M. van Gisbergen, Pleun Hombrink

**Affiliations:** ^1^Department of Hematopoiesis, Sanquin Research and Landsteiner Laboratory, Amsterdam, Netherlands; ^2^Department of Experimental Immunology, Academic Medical Center, Amsterdam, Netherlands; ^3^Renal Transplant Unit, Division of Internal Medicine, Academic Medical Centre, Amsterdam-Zuidoost, Netherlands; ^4^College of Medical and Dental Sciences, Institute of Immunology and Immunotherapy, University of Birmingham, Edgbaston, Birmingham, UK

**Keywords:** Hobit, cytotoxic, CD4^+^ T cells, human cytomegalovirus, human

## Abstract

The T cell lineage is commonly divided into CD4-expressing helper T cells that polarize immune responses through cytokine secretion and CD8-expressing cytotoxic T cells that eliminate infected target cells by virtue of the release of cytotoxic molecules. Recently, a population of CD4^+^ T cells that conforms to the phenotype of cytotoxic CD8^+^ T cells has received increased recognition. These cytotoxic CD4^+^ T cells display constitutive expression of granzyme B and perforin at the protein level and mediate HLA class II-dependent killing of target cells. In humans, this cytotoxic profile is found within the human cytomegalovirus (hCMV)-specific, but not within the influenza- or Epstein–Barr virus-specific CD4^+^ T cell populations, suggesting that, in particular, hCMV infection induces the formation of cytotoxic CD4^+^ T cells. We have previously described that the transcription factor Homolog of Blimp-1 in T cells (Hobit) is specifically upregulated in CD45RA^+^ effector CD8^+^ T cells that arise after hCMV infection. Here, we describe the expression pattern of Hobit in human CD4^+^ T cells. We found Hobit expression in cytotoxic CD4^+^ T cells and accumulation of Hobit^+^ CD4^+^ T cells after primary hCMV infection. The Hobit^+^ CD4^+^ T cells displayed highly overlapping characteristics with Hobit^+^ CD8^+^ T cells, including the expression of cytotoxic molecules, T-bet, and CX3CR1. Interestingly, γδ^+^ T cells that arise after hCMV infection also upregulate Hobit expression and display a similar effector phenotype as cytotoxic CD4^+^ and CD8^+^ T cells. These findings suggest a shared differentiation pathway in CD4^+^, CD8^+^, and γδ^+^ T cells that may involve Hobit-driven acquisition of long-lived cytotoxic effector function.

## Introduction

After activation, CD4^+^ T cells differentiate into various subsets that can be distinguished based on their unique cytokine milieu and transcription factor profile. To date, numerous T helper (T_H_) subsets have been described, including T_H_1, T_H_2, T_H_17, and T follicular helper CD4^+^ T cells (1–3). Classically, CD4^+^ T cells are referred to as cells that exert their helper functions to support other immune cells in their activation and maintenance. For example, CD4^+^ T cells assist B cells in inducing antibody class switching and establishing germinal centers through the secretion of cytokines (4). On the other hand, the capacity to lyse infected target cells, referred to as cytotoxicity, has been attributed to CD8^+^ T cells. However, recently, interest in the cytotoxic capacity of CD4^+^ T cells has been revived. In mice and humans, it has been shown that cytotoxic CD4^+^ T cells play protective roles in cytomegalovirus (CMV) infections (5, 6). While the population of cytotoxic CD4^+^ T cells is less abundant than that of cytotoxic CD8^+^ T cells, cytotoxic CD4^+^ T cells are as capable as their CD8^+^ equivalents in the killing of target cells (7–9).

Early during infection, human CMV (hCMV)-specific CD4^+^ T cells were demonstrated to produce IFNγ and exhibit a T_H_1 phenotype. During the course of infection, these T_H_1-type CD4^+^ T cells acquire a cytotoxic profile, lose CD27 and CD28, and obtain granzyme B (10). Furthermore, these cells gain the ability to lyse infected target cells in an HLA class II-dependent manner (7). The cytotoxic CD4^+^ T cells retained the capacity to produce IFNγ and also co-produced a multitude of other cytokines, including TNFα. Only recently, HLA class II tetramers have become available that allow for the identification and phenotyping of antigen-specific CD4^+^ T cells directly *ex vivo* (11). Using HLA class II tetramers, hCMV-specific CD4^+^ T cells have been described to conform to the effector-like phenotype with high cytotoxic potential. Similar to their cytotoxic CD8^+^ counterparts, the hCMV-specific CD4^+^ T cells contain lytic granules loaded with granzyme B and perforin that mediate lysis of infected target cells. Cytotoxic hCMV-specific CD4^+^ T cells also express CX3CR1, which may direct migration to inflamed endothelium, a major site of hCMV infection (12, 13).

Previously, we have shown that the transcription factor Homolog of Blimp-1 in T cells (Hobit) is upregulated in CD45RA^+^ effector-type CD8^+^ T cells as well as in hCMV-specific CD8^+^ T cells that display the phenotype of CD45RA^+^ effector-type CD8^+^ T cells. We have also demonstrated that Hobit is involved in the transcriptional regulation of effector functions, including the production of IFNγ and granzyme B (14, 15). As the characteristics of cytotoxic CD8^+^ and CD4^+^ T cells strongly overlap, we hypothesized that these cells share a transcriptional program. In search of relevant transcriptional regulators of cytotoxicity in CD4^+^ T cells, we set out to investigate the involvement of Hobit in the regulation of cytotoxic CD4^+^ T cells.

## Results

### Hobit Is Expressed in CD4^+^CD28^−^ Effector-Type T Cells

**Figure 1:**
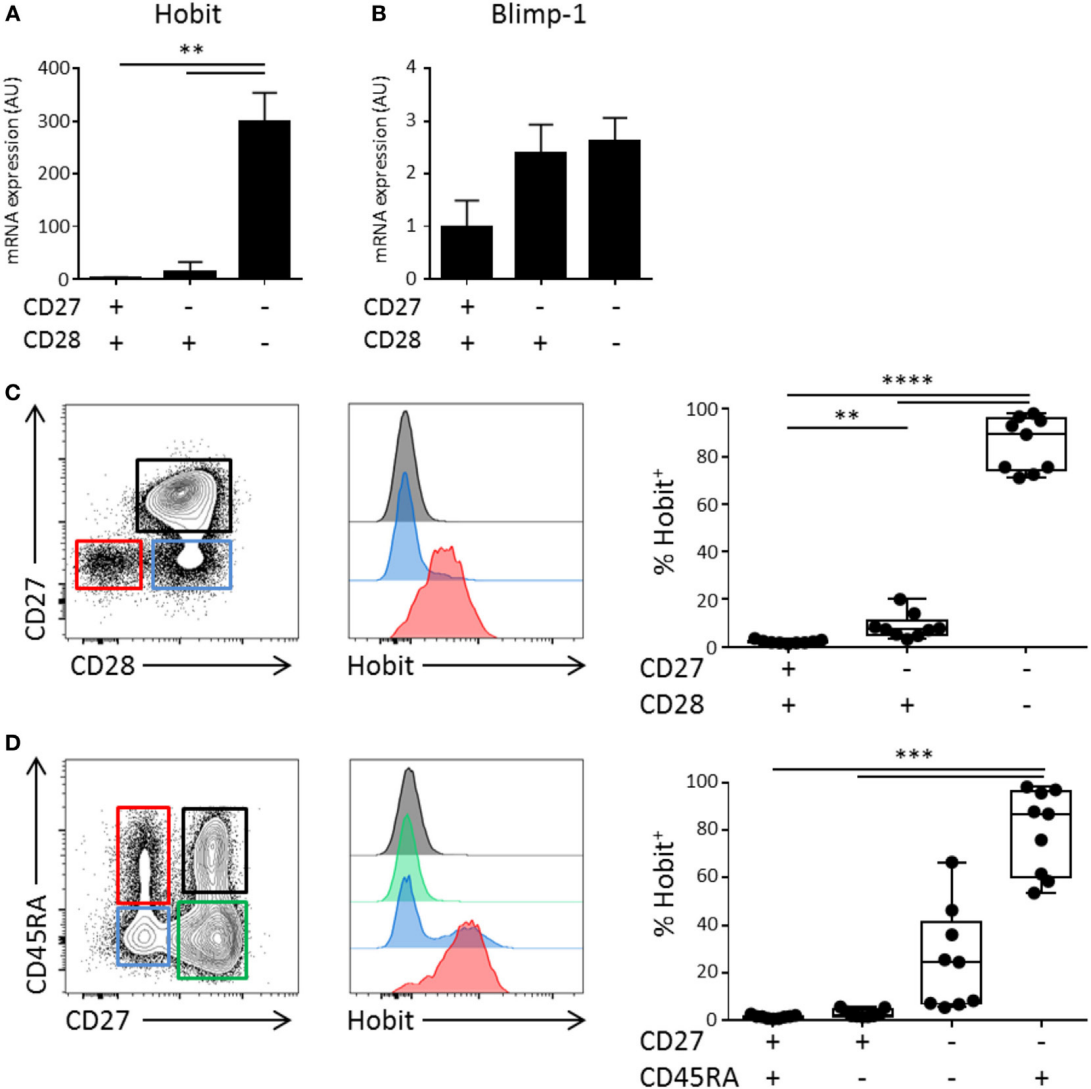
**Hobit is expressed in CD4^+^CD28^−^CD27^−^ effector-type T cells**. Total CD4^+^ T cells can be divided in three fractions based on the expression of CD28 and CD27. **(A,B)** Healthy donor peripheral blood-derived CD4^+^ T cells were sorted based on the expression of CD28 and CD27 and RNA was isolated. Hobit **(A)** and Blimp-1 **(B)** mRNA was measured by qPCR. Values are depicted relative to 18S and calibrated using naïve CD4^+^ T cells. **(C,D)** Hobit expression was identified in different CD4^+^ T cell subsets based on **(C)** the expression of CD27 and CD28 or **(D)** based on the expression of CD45RA and CD27. Representative contour plots are depicted on the left. Stacked histograms (maximum set to 100%) for the color indicated subsets are depicted in the center panels. On the right, the quantification of the percentage of Hobit^+^ cells in the different populations is displayed (*n* = 9). **p < 0.01, ***p < 0.001, ****p < 0.0001; one-way ANOVA with Holm–Sidak’s multiple comparisons test.

Using microarray analysis, we have previously identified Hobit, encoded by *ZNF683*, as one of the most distinctly expressed transcription factors in CD45RA^+^ effector CD8^+^ T cells (16). To investigate the expression pattern of Hobit in CD4^+^ T cell differentiation, we isolated CD4^+^ T cells from the peripheral blood of healthy donors. Effector CD4^+^ T cell differentiation is characterized by the stepwise loss of CD27 and CD28 (10, 12) and, therefore, we sorted CD4^+^ T cells into three populations based on the expression of the co-stimulatory molecules CD28 and CD27. Naïve T cells co-express CD27 and CD28, intermediately differentiated cells downregulate CD27, but not CD28, and terminally differentiated cytotoxic CD4^+^ T cells are characterized by the lack of these two molecules (10, 17, 18). We used qPCR to analyze the expression of Hobit mRNA. Hobit expression was high in cytotoxic CD4^+^CD28^−^CD27^−^ T cells, but nearly absent in CD4^+^CD28^+^CD27^+^ and CD4^+^CD28^+^CD27^−^ T cells (Figure 1A). As Hobit has high homology with Blimp-1, which has been shown to regulate effector T cell differentiation in mice (19), we also assessed the expression of Blimp-1 in the three CD4^+^ T cell populations. In contrast to Hobit, Blimp-1 was equally upregulated in intermediately and terminally differentiated CD4^+^ T cells subsets compared to CD4^+^CD27^+^CD28^+^ T cells (Figure 1B). Reflecting the mRNA analysis, Hobit protein expression was found in terminally differentiated, but not in other CD4^+^ T cells (Figure 1C). Cytotoxic CD4^+^ T cells are described to express either CD45RA or CD45RO (10, 12, 13). Hobit was uniformly expressed by CD4^+^ effector T cells (CD45RA^+^CD27^−^) and by a fraction of effector memory CD4^+^ T cells (CD45RA^−^CD27^−^) (Figure 1D).

### Hobit^+^ CD4^+^ Effector-Type Cells Display a Cytotoxic Profile

**Figure 2:**
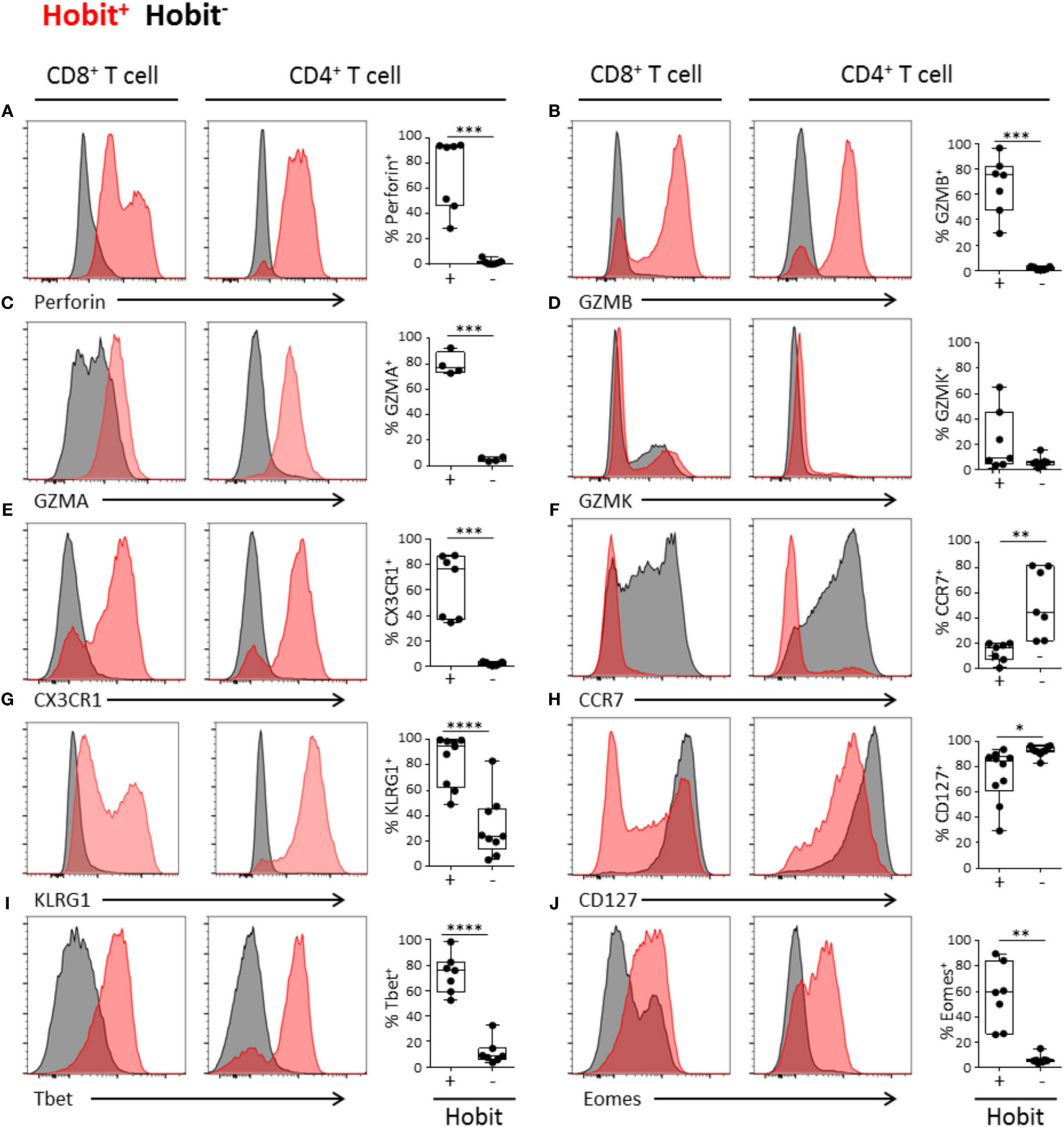
**Hobit^+^ CD4^+^ effector-type cells display a cytotoxic profile**. **(A-J)** Representative histograms (maximum set to 100%) and quantification of the expression of perforin **(A)**, granzyme B **(B)**, granzyme A **(C)**, granzyme K **(D)**, CX3CR1 **(E)**, CCR7 **(F)**, KLRG1 **(G)**, CD127 **(H)**, Tbet **(I)**, and Eomes **(J)** by Hobit^+^ (in red) and Hobit^−^ (in black) CD8^+^ (left column) and CD4^+^ T cells (right column). Box and whiskers plots show the percentage of Hobit^+^ and Hobit^−^ non-naïve CD4^+^ T cells expressing the different molecules (*n* = 4–10). *p < 0.05, **p < 0.01, ***p < 0.001, ****p < 0.0001; paired *T*-test.

In order to further characterize the cytotoxic potential of Hobit^+^ CD4^+^ T cells, we addressed the expression of proteins that are required for the killing of target cells. We found that both perforin and granzyme A and B are highly co-expressed with Hobit, with approximately 75% of Hobit^+^ CD4^+^ T cells expressing perforin, granzyme A, and/or B (Figures 2A–C). In contrast, the cytotoxic molecule granzyme K did not associate with Hobit (Figure 2D). This result is supported by previous reports showing that granzyme K, in contrast to Hobit, is not abundantly expressed in effector CD8^+^ T cells (20). To further investigate the characteristics of Hobit^+^ T cells, we assessed the association of this transcription factor with key properties of cytotoxic T cells. CX3CR1 has been shown to mark cytotoxic CD4^+^ and CD8^+^ T cells and direct them toward fractalkine on activated endothelium (21, 22). It was also recently reported that hCMV-specific CD4^+^ T cells express CX3CR1 (12). We demonstrate that the majority of Hobit^+^ CD4^+^ and CD8^+^ T cells express CX3CR1 (Figure 2E). Of interest is that, *vice versa*, nearly all of the CX3CR1^+^ CD4^+^ and CD8^+^ T cells express Hobit (data not shown).

Previously, CD4^+^ effector T cell differentiation was shown to be associated with the loss of the chemokine receptor CCR7 (7, 10), which mediates the migration of immune cells to secondary lymphoid organs. As Hobit directly suppresses CCR7 expression of lymphocytes in mice (14), we analyzed the expression of this chemokine receptor by human Hobit^+^ T cells. In contrast to the Hobit^−^ fraction, the Hobit^+^ CD4^+^ and CD8^+^ T cells lacked CCR7 expression (Figure 2F). These data suggest that Hobit^+^ T cells do not respond to CCR7-dependent signals that instruct homing to lymphoid organs, in line with the observation that cytotoxic CD4^+^ and CD8^+^ T cells are absent from this compartment (20).

KLRG1 and CD127 are frequently used to differentiate between memory (precursor) cells (KLRG1^−^CD127^+^) and effector cells (KLRG1^+^CD127^−^) (23). In line with effector differentiation, we found that the majority of the Hobit^+^ CD4^+^ T cells and CD8^+^ T cells expressed KLRG1 (Figure 2G). Strikingly, the majority of the Hobit^+^CD4^+^ T cells also expressed CD127, while this molecule was absent on Hobit^+^CD8^+^ T cells (Figure 2H). However, the mean fluorescence intensity of CD127 was lower in Hobit^+^CD4^+^ T cells than in Hobit^−^CD4^+^ T cells (Figure S1 in Supplementary Material). These findings suggest that Hobit^+^CD4^+^ T cells downregulate CD127 similar to effector CD8^+^ T cells, but that the downregulation is incomplete. Homeostatic IL-7 signaling for the maintenance of T cells is mediated by CD127, encoding the IL-7Rα chain (24) indicating that Hobit^+^CD4^+^ T cells may rely on homeostatic IL-7 signaling for their maintenance.

It has been previously demonstrated that the transcription factors T-bet and Eomes are essential for the formation and maintenance of CD8^+^ effector T cells. Hobit expression strongly overlaps with that of T-bet in CD8^+^ effector T cells (25). Similarly, we observed a high degree of overlap between Hobit and T-bet expression in CD4^+^ T cells, with up to 98% of Hobit^+^ cells-expressing T-bet (Figure 2I). Strict co-expression was not observed between Hobit and Eomes. CD4^+^ T cells that expressed Eomes were Hobit^+^ (data not shown), but not all Hobit^+^ CD4^+^ T cells expressed Eomes (Figure 2J). In the case of CD8^+^ T cells, there is substantial co-expression of Hobit and Eomes; however, Hobit^−^ CD8^+^ T cells that express Eomes also exist.

Together, these data demonstrate that Hobit expression identifies CD4^+^ T cells with a cytotoxic profile, suggesting that Hobit is a key mediator of CD4^+^ effector T cell differentiation. The phenotype of Hobit^+^ CD4^+^ T cells parallels that of Hobit^+^ CD8^+^ T cells, suggesting a common transcriptional program of cytotoxicity between the two lineages.

### hCMV-Specific CD4^+^ T Cells Express Hobit

**Figure 3:**
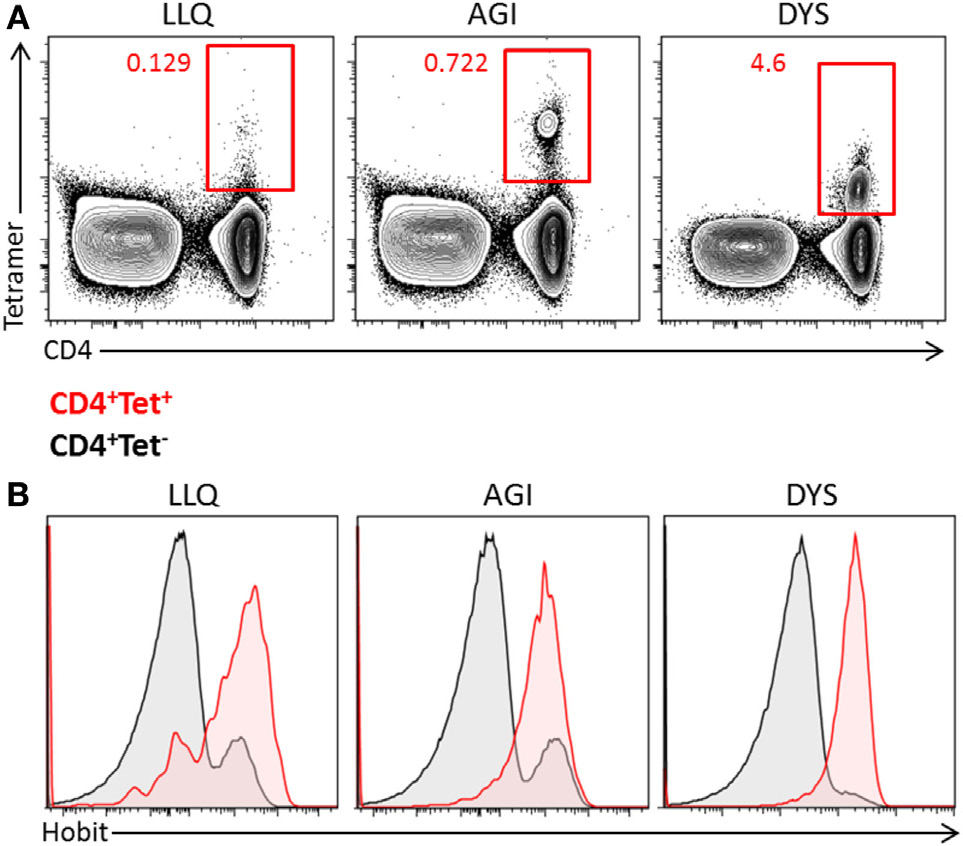
**Human cytomegalovirus (hCMV)-specific CD4^+^ T cells express Hobit**. The expression of Hobit was analyzed in hCMV-specific CD4^+^ T cells using class II tetramers. **(A)** Populations of hCMV-specific CD4^+^ T cells were identified in three donors using three different tetramers, indicated by the first three letters of the peptide sequence (LLQ, AGI, and DYS). The contour plots show total PBMCs with CD4 on the *x*-axis and tetramer on the *y*-axis. **(B)** The histograms show the expression of Hobit on CD4^+^ tetramer^+^ T cells (red) and CD4^+^ tetramer^−^ T cells (black) of three donors.

As hCMV induces the differentiation of cytotoxic CD4^+^ T cells, we analyzed the expression of Hobit in hCMV-specific CD4^+^ T cells of hCMV-seropositive donors using HLA class II tetramers specific for various hCMV epitopes. The frequencies of hCMV-specific CD4^+^ T cells varied between 0.1 and 4.6% of total T cells in the three different individuals (Figure 3A), the hCMV-specific CD4^+^ T cells recognizing the analyzed epitopes all expressed Hobit to a substantial degree (Figure 3B), similar to hCMV-specific CD8^+^ T cells, as we have previously shown (25). Thus, the hCMV-driven differentiation of cytotoxic CD4^+^ T cells includes the upregulation of Hobit expression.

### Hobit^+^ CD4^+^ T Cells Increase over Time during Primary hCMV Infection *In Vivo*

**Figure 4:**
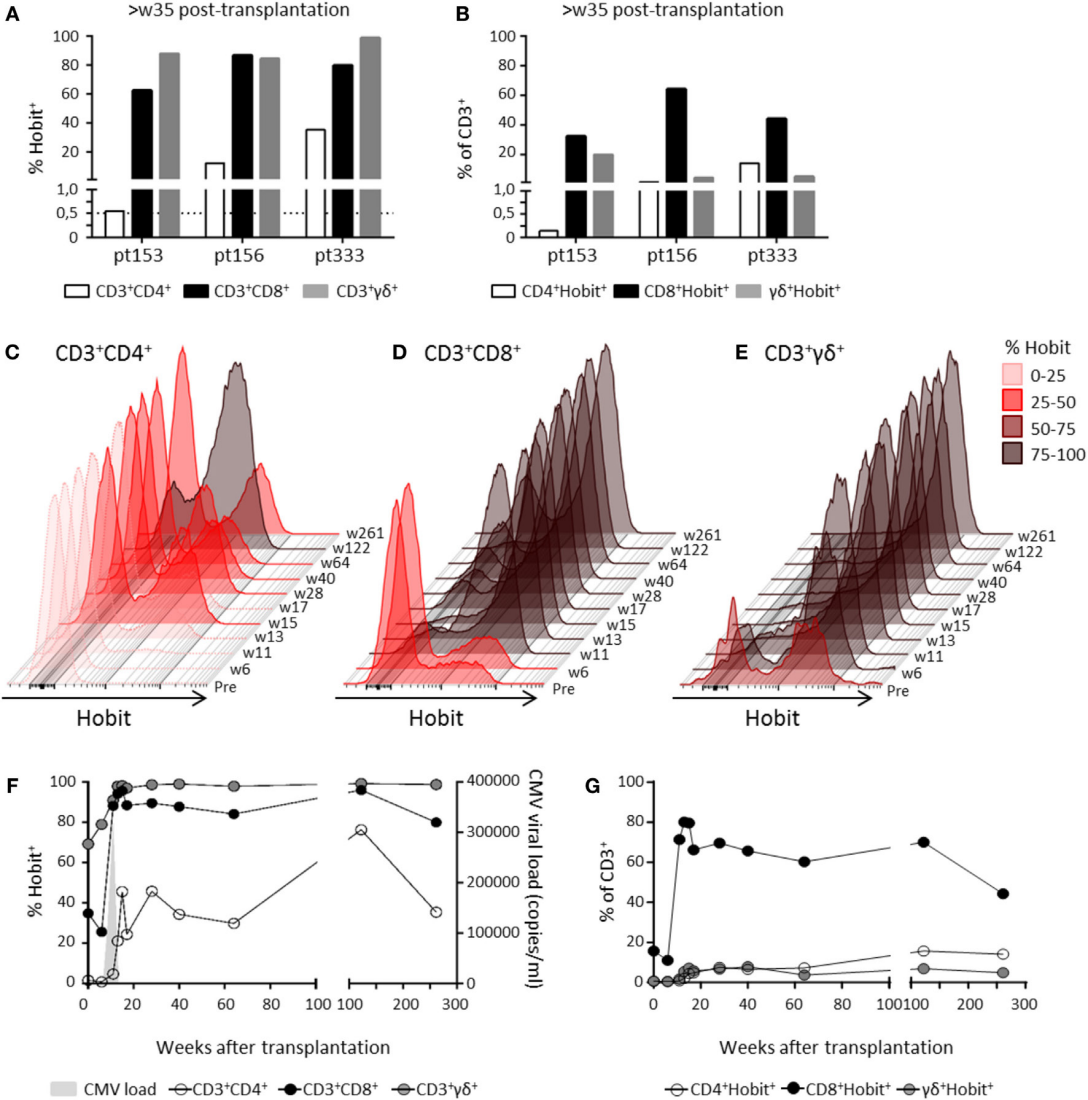
**Hobit^+^ CD4^+^ T cells increase over time during primary human cytomegalovirus (hCMV) infection *in vivo***. The induction of Hobit expression in CD4^+^, CD8^+^, and γδ^+^ effector type T cells was followed over the course of primary hCMV infection. **(A)** Frequency of Hobit^+^ cells of CD4^+^, CD8^+^, and γδ^+^ T cells within the population and **(B)** the abundance of Hobit^+^CD4^+^, Hobit^+^CD8^+^, and Hobit^+^γδ^+^ in the total CD3^+^ T cells in three hCMV-seronegative recipients (pt153, pt156, and pt333) was analyzed at more than 35 weeks after kidney transplantation from a hCMV-seropositive donor. The dotted line in **(A)** indicates the percentage of cytotoxic CD4^+^ T cells minimally induced by hCMV infection. **(C–E)** The overlayed histograms (maximum set to 100%) demonstrate the kinetics of Hobit expression in total **(C)** CD4^+^, **(D)** CD8^+^, and **(E)** γδ^+^ T cells over the course of primary hCMV infection in pt333. On the right, the sampling time points are indicated in weeks (w) posttransplantation. The color code indicates the percentage of Hobit^+^ cells according to the depicted key. **(F)** The line graph shows the induction of Hobit expression (left *y*-axis) in CD4^+^ (white), CD8^+^ (black), and γδ^+^ (gray) T cells after kidney transplantation in pt333. The *x*-axis shows the weeks posttransplantation. Viral loads (determined by qPCR and in light gray) is plotted on the right *y*-axis as copies of hCMV per milliliter of blood. **(G)** The abundance of the Hobit^+^CD4^+^, Hobit^+^CD8^+^, and Hobit^+^γδ^+^ in the total CD3^+^ T cells is shown over the course of the hCMV infection for pt333.

To investigate Hobit expression after primary hCMV infection, we analyzed CD4^+^ T cells in samples derived from three hCMV-seronegative recipients who had received hCMV-positive kidney transplants at least 35 weeks ago. As hCMV infection is known to also induce the expansion of CD8^+^ and γδ^+^ effector T cells (26, 27), we analyzed these T cell lineages in parallel to the CD4^+^ T cells. In one patient (pt 153), Hobit expression within CD4^+^ T cells and the abundance of Hobit^+^ CD4^+^ T cells remained low compared to the massive expansion of Hobit^+^ γδ^+^ and CD8^+^ T cells (Figures 4A,B). Although the CD4^+^ response was low, it still passed the threshold of 0.5% cytotoxic CD28^−^CD4^+^ T cells previously shown to be the minimal indication of hCMV infection (10). In contrast, the other two patients (pt 156 and 333) showed a substantial expansion of Hobit^+^ cells within the CD4^+^ T cell compartment as well as the CD8^+^ and γδ^+^ T cell compartments (Figures 4A,B). We analyzed the kinetics of the expansion of the three T cell lineages further (Figures 4C–G; Figure S2 in Supplementary Material). The accumulation of Hobit^+^ CD4^+^ T cells only started after clearance of viral infection between weeks 11 and 13 (Figures 4C,F). After about 15 weeks, the Hobit-expressing CD4^+^ T cell population stabilized at around 40% of CD4^+^ T cells and 10% of total lymphocytes (Figures 4F,G). These findings closely correspond with the kinetics of the emergence of a CD4^+^CD28^−^ population after hCMV infection (10). Furthermore, the kinetics of the CD4^+^ effector response appeared similar to that of the effector CD8^+^ T cell and γδ^+^ T cell response, except that the overall magnitude was lower for the response of the CD4^+^ and γδ^+^ T cells than for that of the CD8^+^ T cells (Figures 4C–G).

### Hobit Identifies CD4^+^ Effector-Type T Cells during Primary hCMV Infection *In Vivo*

**Figure 5:**
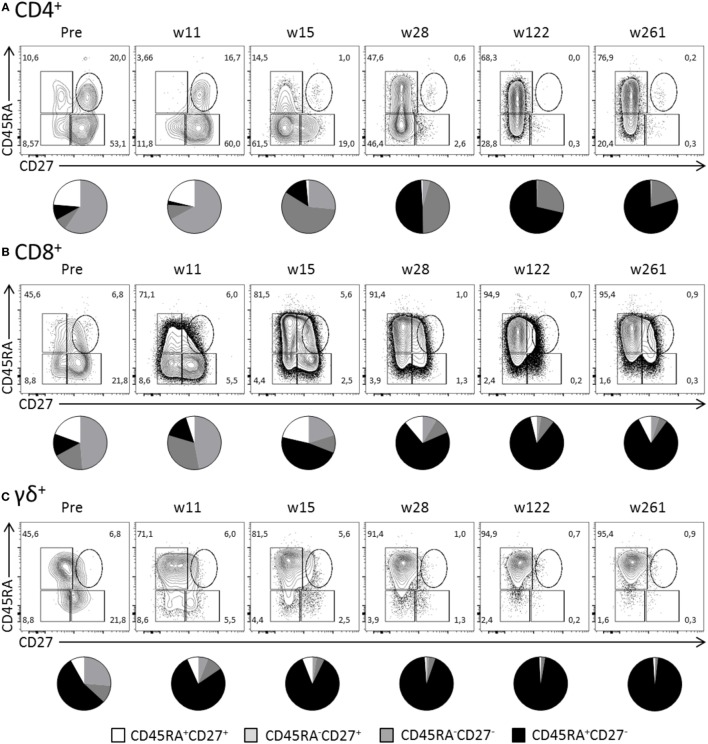
**Hobit identifies expanded CD4^+^ effector-type T cells during primary human cytomegalovirus (hCMV) infection *in vivo***. The distribution of CD45RA/CD27 expression by Hobit^+^ **(A)** CD4^+^, **(B)** CD8^+^, and **(C)** γδ^+^ T cells was characterized over the course of hCMV infection for pt333. Top panels show contour plots of CD45RA/CD27 of Hobit^+^ T cells for the indicated time points after transplantation (weeks). Lower panels show pie charts representing the distribution of CD45RA/CD27 expression by Hobit^+^ T cells of the three lineages.

Surface expression of CD45RA and CD27 defines naïve (CD45RA^+^CD27^+^), memory (CD45RA^−^), and effector (CD45RA^+^CD27^−^) T cells. As expected during the latency stage of infection (week 28 and onward), the majority of Hobit^+^ CD4^+^ T cells expressed an effector phenotype (Figure 5A). In contrast, pretransplant and at early time points posttransplant, Hobit^+^ CD4^+^ T cells are mainly characterized by a memory phenotype. The Hobit^+^ CD4^+^ T cells first downregulate CD27 at around week 11, followed by the upregulation of CD45RA at around week 15, thereby completing the acquisition of the effector phenotype. Similar downregulation of CD27 preceding the upregulation of CD45RA was found for Hobit^+^ CD8^+^ and Hobit^+^ γδ^+^ T cells, albeit with faster kinetics (Figures 5B,C). Importantly, the phenotypic composition of the Hobit^−^ CD4^+^, CD8^+^, and γδ^+^ T cell population stably represented naïve and memory T cells throughout the sampling period (Figure S3 in Supplementary Material), suggesting that the events in T cell differentiation during the course of hCMV infection are restricted to the Hobit^+^ cells. Taken together, these data suggest that Hobit expression precedes the development of terminally differentiated effector T cells, which supports an instructive role of the transcription factor in the acquisition of the effector profile.

### Transcriptional Programming of Cytotoxic CD4^+^ T Cells

**Figure 6:**
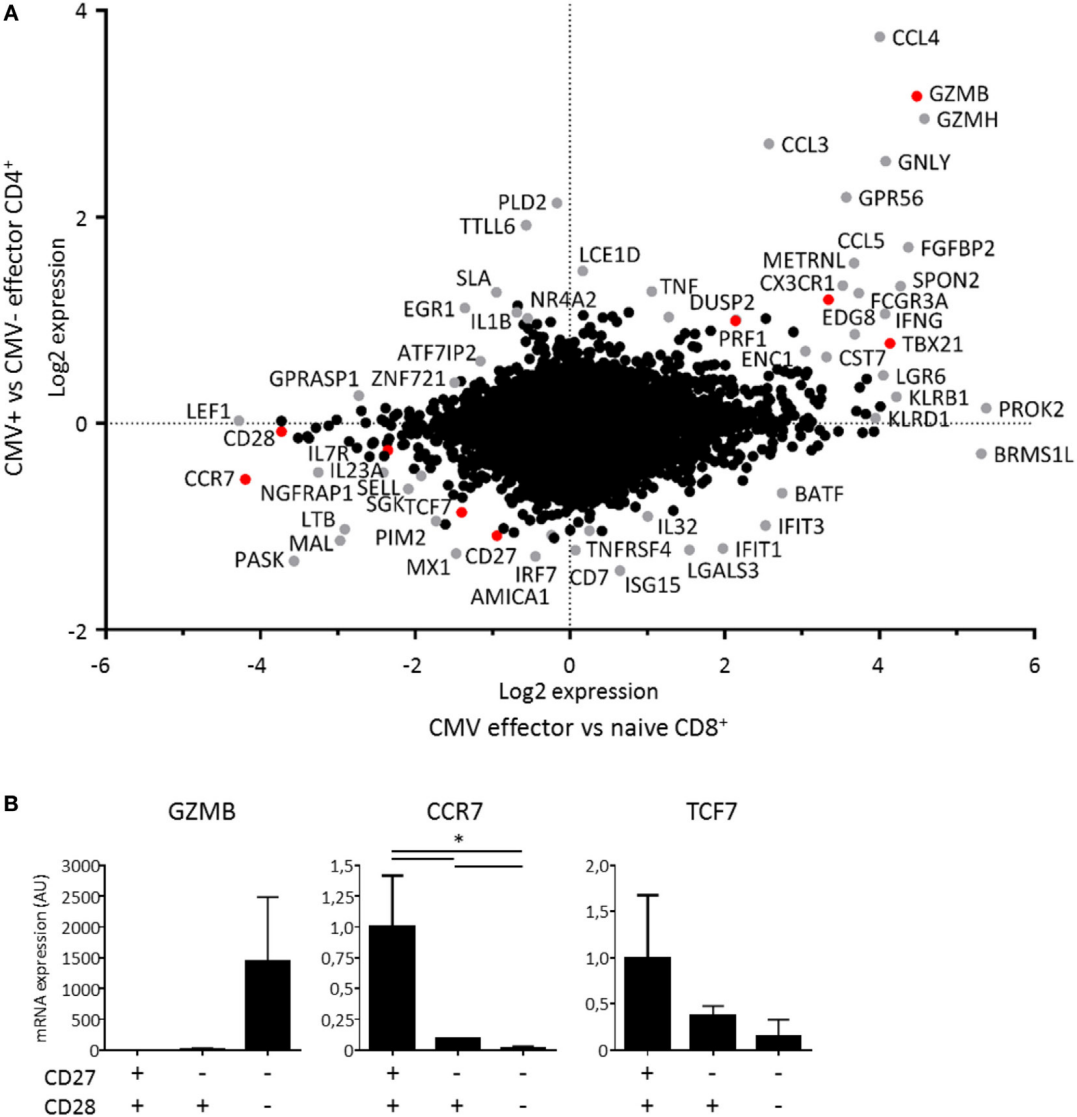
**Transcriptional profile of cytotoxic CD4^+^ T cells**. The transcriptional profile of cytotoxic CD4^+^ T cells was determined by comparing the signature of human cytomegalovirus (hCMV)-specific CD4^+^ T cells to effector CD8^+^ T cells during a primary hCMV infection. **(A)** The dot plot shows the genes that are up- and downregulated by hCMV-specific CD4^+^ T cells compared to CMV-effector CD4^+^ T cells on the *y*-axis and the genes that are up- and downregulated by effector CD8^+^ T cells in a primary hCMV infection compared to naive CD8^+^ T cells on the *x*-axis. The changes shown are on a log2 scale on both axes. The differentially regulated genes in either comparison are depicted in grey except for the genes described at protein level in Hobit^+^CD4^+^ T cells in Figures 1 and 2, which are shown in red. **(B)** The specific target genes of Hobit that were differentially expressed, GZMB, CCR7, and TCF7 were confirmed by qPCR in sorted CD4^+^ T cell subsets (CD28^+^CD27^+^, CD28^+^CD27^−^, CD28^−^CD27^−^). The values are depicted as relative to 18S and calibrated to naïve CD4^+^ T cells. For the qPCR data, *n* = 4. *p < 0.05; one-way ANOVA with Holm–Sidak’s multiple comparisons test.

The parallels between cytotoxic CD4^+^ and CD8^+^ T cells prompted us to compare the transcriptional profiles of these cells. Therefore, we made a comparison of the microarray data on hCMV-specific CD4^+^ T cells (12) with microarray data on CD8^+^ T cells during a primary hCMV response (16). We found substantial overlap between the transcriptional profiles of the effector CD4^+^ and CD8^+^ T cells (Figure 6A). The CD4^+^ microarray did not include the probe set for ZNF683 (Hobit), which excluded Hobit from the comparison. Genes encoding perforin, granzyme B, CX3CR1, Tbet, CD27, and CCR7 (highlighted in red) were similarly regulated in cytotoxic CD4^+^ and CD8^+^ T cells (Figure 6A), in contrast to genes encoding the IL-7R and CD28 (also highlighted in red). These findings largely substantiate our results as the products of these genes except for the IL-7R associated with Hobit in both CD4^+^ and CD8^+^ T cells (Figures 1 and 2).

Within the shared up- and downregulated genes, we also identified known targets of Hobit that include GZMB, CCR7, and TCF7 (14, 15). To confirm the microarray data, we sorted CD28^−^CD27^−^, CD28^+^CD27^−^, and CD28^+^CD27^+^ CD4^+^ T cells and performed qPCRs for these Hobit targets (Figure 6B). We found that GZMB was exclusively expressed in the CD28^−^CD27^−^ subset, while mRNA expression of CCR7 and TCF7 was low in this subset, intermediate in the CD28^+^CD27^−^ subset, and high in the CD28^+^CD27^+^ subset. These findings are in line with a role for Hobit in the regulation of the transcriptional program of cytotoxic CD4^+^ T cells.

## Discussion

Transcription factors initiate differentiation processes by instructing lineage-specific gene programs. Therefore, they are interesting candidates in the identification of cell subsets through their expression profiles that are strongly associated with unique cell populations. In this report, we have identified the transcription factor Hobit as a marker of cytotoxic CD4^+^ T cells in humans. Hobit is expressed by all cytotoxic CD4^+^ T cells, but absent from other CD4^+^ T cell populations in peripheral blood including regulatory T cells, as described previously (25). Expression of Hobit in CD4^+^ T cells on its own was sufficient to adequately identify cytotoxic CD4^+^ T cells, suggesting that the transcription factor is a useful alternative for the identification of these cells. The advantage of Hobit over the classical definition of cytotoxic CD4^+^ T cells using CD27 and CD28 is that in contrast to these co-stimulatory molecules, Hobit acts as a positive marker, which improves the accuracy of the typing of these cytotoxic CD4^+^ T cells. Furthermore, as Hobit expression precedes development of effector-type CD4^+^ T cells, Hobit may allow for earlier recognition of the expanding cytotoxic CD4^+^ T cell population.

Hobit is also expressed outside of the CD4^+^ T cell lineage in CD45RA^+^ effector CD8^+^ T cells and CD56^dim^ NK cells (25). Interestingly, here, we showed that the spectrum of Hobit^+^ lymphocytes also includes effector γδ^+^ T cells. Similar to Hobit^+^ CD4^+^ T cells, these Hobit^+^ populations within the CD8^+^ T cell, γδ^+^ T cell, and NK cell lineages are cytotoxic lymphocytes that maintain constitutive protein expression of granzyme B and perforin. The overlap in the expression profiles between the cytotoxic populations of lymphocytes extends beyond the expression of cytotoxic molecules and includes the downregulation of CD27, CD28, and CCR7 and the upregulation of CX3CR1 and T-bet. The similarity in phenotypes suggests that these cytotoxic lymphocytes are under the control of overlapping transcriptional programs. Currently, direct evidence for a role of Hobit in the transcriptional regulation of cytotoxicity in human lymphocytes is not available. Given that we have previously shown that Hobit is essential for the upregulation of granzyme B expression in NKT cells in mice (15), it is conceivable that Hobit also instructs the cytotoxic program of human lymphocytes. In support of an instructive role of Hobit in cytotoxic CD4^+^ T cells, we found that granzyme B and other target genes of Hobit, including CCR7 and TCF7, were up- or downregulated at the transcript level in these cells. The co-expression of T-bet and Hobit, and to a lesser extent Eomes, suggests that these transcription factors may concordantly contribute to the transcriptional regulation of the cytotoxic program in human cytotoxic lymphocytes.

CD4^+^ T cells with a cytotoxic phenotype arise after primary infection with hCMV in humans (7, 10, 28). Peptide restimulation, proliferation, and tetramer studies have also identified hCMV-specific cells within the population of cytotoxic CD4^+^ T cells (7, 10, 12). It has been suggested that cytotoxic CD4^+^ T cells have arisen to provide an additional layer of HLA class II-dependent protection in response to the immune evasive strategies of the hCMV virus that are targeted at CD8^+^ T cell and NK cell-driven immunity (7). We describe that the CD4^+^ T cells that accumulate after primary hCMV infection in transplant patients express Hobit, suggesting that upregulation of Hobit is an integral part of the cytotoxic CD4^+^ T cell response against hCMV. Furthermore, our class II tetramer studies directly showed that hCMV-specific CD4^+^ T cells nearly uniformly express Hobit. At this time, it remains unclear, which signals drive the expression of Hobit in cytotoxic CD4^+^ T cells after hCMV infection. Previously, we have described that T-bet and IL-15 induce Hobit expression in murine CD8^+^ T cells (14). Consistent with these findings, T-bet and Hobit are strongly co-expressed in human CD4^+^ T cells and, as described previously, in human CD8^+^ T cells (25). Antigenic stimulation downregulates expression of Hobit in NKT cells (15). Interestingly, the Hobit^+^CD4^+^ T cell response demonstrated relative similar kinetics as the CD8^+^ and γδ^+^ T cell responses after resolution of primary hCMV infection, suggesting that antigenic stimulation also antagonizes Hobit expression in human T cells. Thus, it is plausible that, after viral clearance, T-bet mediates the upregulation of Hobit expression in CD4^+^ T cells.

Similar to CD45RA^+^ effector CD8^+^ T cells, cytotoxic CD4^+^ T cells are long-lived and highly effective in the elimination of infected target cells (7). The combination of these useful properties suggests that cytotoxic CD4^+^ T cells may be of potential interest in adoptive transfer strategies for the treatment of patients. However, many hurdles need to be overcome before cytotoxic CD4^+^ T cells can be used in cellular therapy. CD4^+^ T cells appear fragile after isolation, do not robustly expand after TCR stimulation, and may be difficult to maintain on homeostatic cytokines such as IL-7 due to low expression of CD127. Therefore, a better understanding of the role of Hobit in the differentiation pathway of these cells may be instrumental to improve protocols for the formation and expansion of cytotoxic CD4^+^ T cells.

## Materials and Methods

### Subjects

PBMCs were isolated from buffy coats of healthy donors supplied by Sanquin Blood Supply Foundation. The CMV status of the healthy donors is unknown. We also received samples longitudinally from one kidney transplant patient (patient 156) who was seronegative for Epstein–Barr virus (EBV) and hCMV prior to the transplant. We also received samples from two patient kidney transplant patients (patient 153 and 333) who were EBV seropositive but hCMV seronegative prior to the transplant. Two of the patients (patient 153 and 333) developed a primary hCMV infection while the other (patient 156) developed a primary EBV and hCMV infection after receiving a kidney from an EBV^+^ and hCMV^+^ donor. The patients received immunosuppressive treatment, including prednisolone, cyclosporine A, and mycophenolate mofetil.

### Ethics Statement

Written informed consent was given by all of the patients. The Amsterdam Medical Center Medical Ethical Committee approved the study according to the Declaration of Helsinki.

### Isolation of Mononuclear Cells from Peripheral Blood

PBMCs were isolated from heparinized peripheral blood samples with standard density gradient centrifugation method and cryopreserved until further analysis.

### Flow Cytometric Cell Sorting

For the analysis of Hobit, Blimp1, GZMB, CCR7, and TCF7 mRNA, CD3^+^CD4^+^CD28^+^CD27^+^, CD3^+^CD4^+^CD28^+^CD27^−^, and CD3^+^CD4^+^CD28^−^CD27^−^ T cells were isolated using flow cytometric sorting for CD3, CD4, CD28, and CD27 using FACS Aria (BD).

### Quantitative PCR

RNA was isolated from the sorted samples using Invisorb RNA isolation kit (Invitek) or Trizol reagent (Invitrogen). cDNA was synthesized using RevertAID H Minus Reverse Transcriptase (Thermo Scientific) and random primers (Invitrogen) or poly dT oligos (Invitrogen). qPCR analysis was performed using Power SYBR Green (Applied Biosystem) with StepOnePlus Real-Time PCR system (Applied Biosystem). The following primers were used: Hobit (forward: 5′-CATATGTGGCAAGAGCTTTGG-3′, reverse: 5′-AGAGCTTCACTCAACTTGCC-3′), Blimp-1 (forward: 5′-CAACAACTTTGGCCTCTTCC-3′, reverse: 5′-GCATTCATGTGG CTTTTCTC-3′), GZMB (forward: 5′- TGCGAATCTGACTTACGCCAT-3′, reverse: 5′- GGAGGCATGCCATTGTTTCG-3′), CCR7 (forward: 5′- CAGCCTTCCTGTGTGGTTTT-3′, reverse: 5′- AAATGACAAGGAGAGCCACC-3′) and TCF7 (forward: 5′-AGAGAGAGAGTTGGGGGACA-3′, reverse: 5′-TCTGCTCATGCATTACCCAC-3′), and 18S (forward: 5′-GGACAACAAGCTCCGTGAAGA-3′, reverse: 5′- CAGAAGTGACGCAGCCCTCTA-3′). Values are depicted as relative to 18S and calibrated to naïve CD4^+^ T cells.

### Flow Cytometry Analysis

PBMCs were labeled with different combinations of the following antibodies: CD4 BUV737 (BD, clone SK3), CD8 BV786 (BD, clone RPA-T8), CD8 BUV805 (BD, clone SK1), CD3 V500 (BD, clone UCHT1), CD3 eVolve605 (eBioscience clone OKT3), CD27 APC-eFluor780 (eBioscience, clone), CD27 BV510 (Biolegend, clone O323), CD45RA BV650 (BD, clone HI100), CD45RA Qdot655 (Invitrogen, clone MEM-56), CD28 PE-Cy7 (BD, clone 28.2), CX3CR1 APC (eBioscience, clone 2A9-1), CCR7 BV421 (Biolegend, clone G043H7), CD127 BV421 (Biolegend, clone A019D5). Near-IR fixable dye (Invitrogen) was used to exclude dead cells from the analysis. For intracellular staining, the following antibodies were used: Hobit IgM (BD, clone Sanquin-Hobit/1), Eomes eFluor660 (eBioscience, clone WD1928), Tbet BV421 (Biolegend, clone 4B10), Granzyme B AF700 (BD, clone GB11), Perforin FITC (eBioscience, clone dG9), Perforin PE (eBioscience, clone B-D48), Granzyme K PE (Immunotools, clone 24C3), Granzyme K FITC (Immunotools, clone 24C3). To stain for Hobit IgM, a secondary anti-IgM labeled with PE or FITC was used. The cells were labeled according to manufacturer’s instructions. For the intracellular staining, the cells were fixed and permeabilized using the Foxp3/Transcription Factor Staining kit (eBioscience). The samples were measured in PBS 0.5% FCS with a LSR Fortessa (BD). The analysis was done using FlowJo Version 10 software.

### Class II Tetramer Analysis

The class II tetramers used in this study and the staining protocol were previously described by Pachnio et al (12). Briefly, three tetramers were used against three different hCMV epitopes; gB-derived epitope DYSNTHSTRYV (HLA-DRB1*07:01) and pp65-derived epitopes AGILARNNLVPMVATV (HLA-DRB3*02:02) and LLQTGIHVRVSQPSL (HLA-DQB1*06:02).

### Statistics

To determine the significance of our results, we used the paired *T*-test or one-way ANOVA and Holm–Sidak’s multiple comparisons test using GraphPad Prism 6. A *p*-Value of less than 0.05 was considered statistically significant (*p < 0.05, **p < 0.01, ***p < 0.001, ****p < 0.0001).

## Author Contributions

AO, RL, KG, and PH designed the project and experiments. AO, FB, ER, NK, KH, and JZ performed the experiments. All authors contributed to the interpretation and discussion of the data. AO, KG, and PH wrote the manuscript. All authors read and approved the manuscript.

## Conflict of Interest Statement

The authors declare that the research was conducted in the absence of any commercial or financial relationships that could be construed as a potential conflict of interest.
