# Supplementary material for: The Transcription Factor Hobit Identifies Human Cytotoxic CD4+ T Cells
Source: Front Immunol. 2017 Mar 24;8:325. doi: 10.3389/fimmu.2017.00325 (PMC5364140; doi:10.3389/fimmu.2017.00325)
Supplement: Supplementary file 1 [file data_sheet_1.pdf]

Supplementary Material

The transcription factor Hobit identifies human cytotoxic CD4$^+$ T cells

Anna E. Oja[#1], Felipe A. Vieira Braga[#1], Ester B. M. Remmerswaal[2,3], Natasja A. M. Kragten[1], Kirsten M.L. Hertoghs[2], Jianmin Zuo[4], Paul Moss[4], René A.W. van Lier[1], Klaas P. J. M. van Gisbergen[1,2##*], Pleun Hombrink[1##*]

*** Correspondence:**

Dr. Klaas P.J.M. van Gisbergen and Dr. Pleun Hombrink
k.vangisbergen@sanquin.nl and p.hombrink@sanquin.nl

1 Supplementary Figures

1.1 Supplementary Figure 1

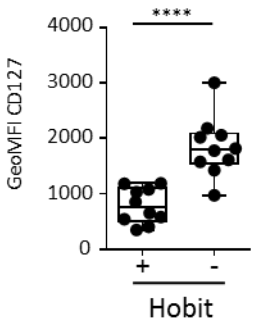

Suppl Figure 1: **Geometric mean fluorescence intensity of CD127 by Hobit$^+$ and Hobit$^-$ CD4$^+$ T cells.** The geometric mean fluorescence intensity of CD127$^+$ cells was quantified for the Hobit$^+$ and Hobit$^-$ non-naïve CD4$^+$ T cells. $n = 10$. **** $p < 0.0001$; paired T test.

1.2 Supplementary Figure 2

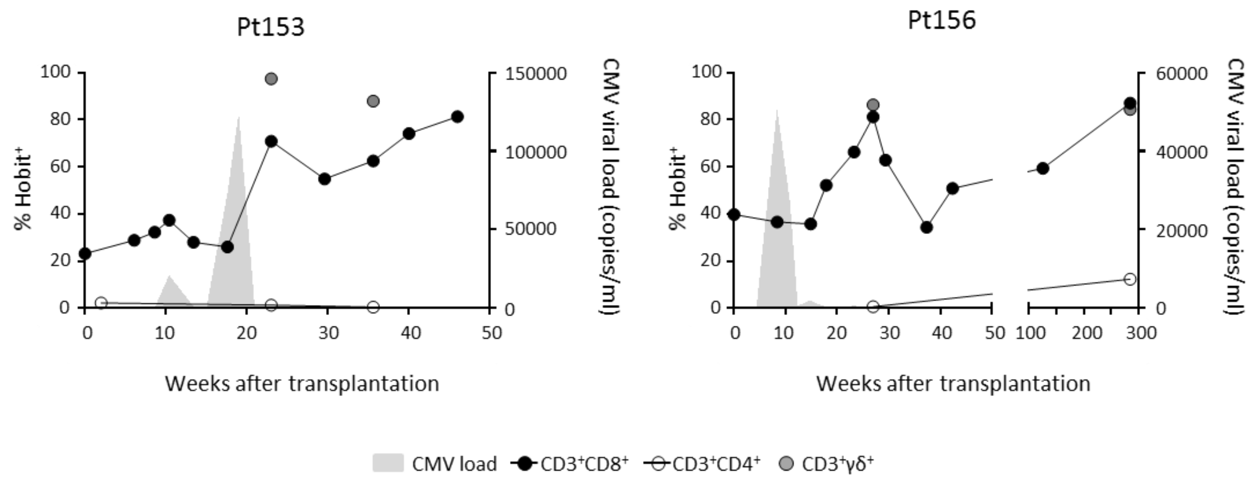

Suppl Figure 2: **Expansion of Hobit$^+$ T cells over time after primary hCMV infection.** The line graphs show the time course analysis of Hobit expression in CD4$^+$, CD8$^+$, and γδ$^+$ T cells after kidney transplantation in pt153 and pt156. Left y-axis depicts the percentage of Hobit$^+$ cells within the CD4$^+$ (white), CD8$^+$ (black), and γδ$^+$ (grey) T cell populations. The CD4$^+$ and γδ$^+$ T cells were analyzed at three and two time points, respectively. The CD8$^+$ expansion was measured more frequently. Viral loads (determined by qPCR) (light grey) are depicted on the right y-axis as copies of hCMV per ml of blood.

1.3 Supplementary Figure 3

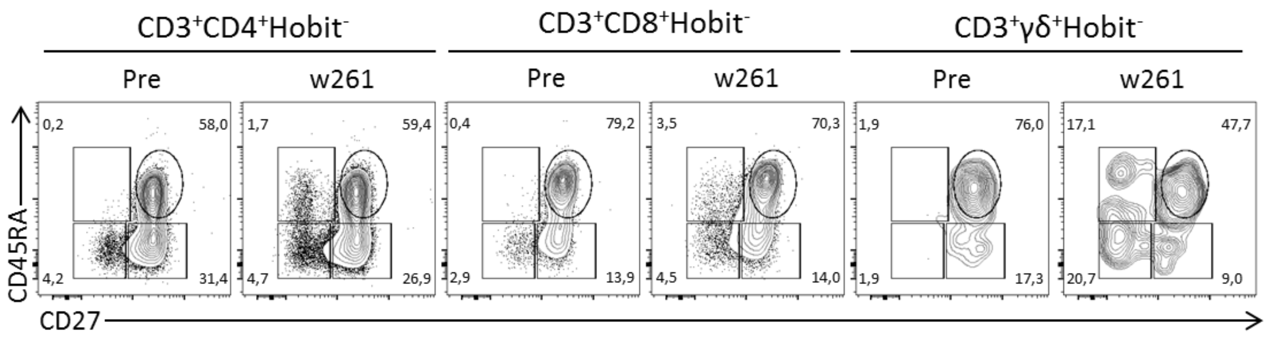

Suppl Figure 3: **Phenotype of Hobit$^-$ T cells before and after primary hCMV infection.** Distribution of CD45RA/CD27 expression, shown with contour plots, by Hobit$^-$ CD4$^+$, CD8$^+$ and γδ$^+$ T cells was characterized prior to transplantation and 261 weeks post transplantation of hCMV infection for pt333.
